# Bœnninghausen and Lippe

**Published:** 1890-01

**Authors:** Edmund J. Lee

**Affiliations:** Philadelphia


### BŒNNINGHAUSEN AND LIPPE.

EDMUND J. LEE, M. D., PHILADELPHIA.

In an article in the *Medical Advance*, a year or so ago, under the above title, a false impression is unintentionally given of the late Dr. Lippe as a homœopathic prescriber; we read: "But in some particulars his (Lippe's) methods differed so widely from those of Bœnninghausen that a comparison can scarcely do justice to either. The former was slower, more cautious, and, as they say, 'dug out the remedies' by hard work, and like all workers of that kind, made fewer mistakes." Further, we read: "The one (L.) was often found exhibiting flashes of true genius in his searches for, and seizing upon the true remedy for his case; the other (B.), with utmost coolness and deliberation, sought for the secret of relationship between sicknesses and their causation, which when once struck was pursued with the pertinacity and unwearied persistency of the sleuth hound, till he found the true remedy for his case, and this he did with a certainty which came near to uniform success." As we have said, these opinions give a false impression, especially to those who did not know Dr. Lippe personally. This impression has already borne fruit, for at the meeting of the I. H. A., of 1888, a speaker alluded to Dr. Lippe as "a flash prescriber." We reiterate, this view of Dr. Lippe's success is misleading No physician can succeed in the practice of homœopathic medicine unless he studies and studies hard at that; no one knew this better than the late Dr. Ad. Lippe. He was endowed with the gift for drug analysis and a quickness of perception in discerning the symptoms of his patients which were the indicators for choosing his drug. The only other "genius" Dr. Lippe possessed was a disposition to work hard and a profound admiration for the *Organon*. There are few men of even moderate ability who cannot achieve all of Dr. Lippe's "genius" if they work as hard as he did and in the right way. Bœnninghausen was undoubtedly a grand prescriber, and it is an honor for any one to be compared with him; but we doubt if he, or any other physician, ever studied out his cases with more unvaried care than did the late Dr. Lippe. No flashes of genius could have enabled one to cure the many chronic cases which he cured; diligent, careful work is required to gain such results. The necessity for this diligent study of the *Materia Medica* was impressed upon Dr. Lippe by his preceptor, the late Dr. Wesselhœft; often have we heard him tell how his preceptor made him thumb his *Materia Medica.*

Often has the writer heard Dr. Lippe say, in speaking of different physicians, "he does not study his cases thoroughly," or make some such remark, indicating the importance he attached to such study. The writer was once associated with Dr. Lippe in the care of a very difficult case, in which he has frequently known him to spend hour after hour patiently conning the *Materia Medica* (this, too, after nearly forty years spent at such study). Many a time would he say, "Come over to-night and we will study the case over;" frequently have we asked his advice; before replying he would almost invariably take down a book or two to look up the remedy desired. Dr. Ad. Lippe was in no sense "a flash prescriber."

Genius has been defined as an unlimited capacity for hard work; this genius Dr. Lippe had and used it diligently (and in the right way) for forty odd years. Was it then any wonder that he could occasionally prescribe quickly? He never prescribed until he felt sure of his remedy, and though he was quick in seeing the peculiar features of his patient's history, and rapid in selecting his remedy, he was never so quick as to be termed

*careless.* There is a distinction to be noted here; one man may spend hours on a case and then make a careless prescription; another with better trained mind may prescribe in a few minutes and make a very careful, accurate prescription.

We do not pretend to affirm that Dr. Lippe did not make mistakes; very probably he would have been the last man to deny legions of them; but we do believe that as a homœopathic prescriber neither Boenninghausen nor Hahnemann himself would suffer in comparing clinical results with their great admirer—Adolph Lippe.

